# Analysis of *In Vivo* Plant Volatiles Using Active Sampling and TD-GC×GC-TOFMS

**DOI:** 10.3390/metabo14110623

**Published:** 2024-11-14

**Authors:** Sheri A. Schmidt, Ewenet Yemane Mesfin, Chaminda De Silva Weeraddana, A. Paulina de la Mata, Alejandro C. Costamagna, James J. Harynuk

**Affiliations:** 1 Department of Chemistry, University of Alberta, Edmonton, AB T6G 2R3, Canada; saschmid@ualberta.ca (S.A.S.); mesfin@ualberta.ca (E.Y.M.); delamata@ualberta.ca (A.P.d.l.M.); 2 The Metabolomics Innovation Centre, Edmonton, AB T6G 2R3, Canada; 3 Department of Entomology, University of Manitoba, Winnipeg, MB R3T 2N2, Canada; chaminda.weeraddana@umanitoba.ca (C.D.S.W.); ale.costamagna@umanitoba.ca (A.C.C.)

**Keywords:** *in vivo* active sampling, comprehensive two-dimensional gas chromatography, plant volatiles, thermal desorption (TD), volatile organic compounds (VOCs)

## Abstract

**Background:** Plants constantly produce primary and secondary metabolites, and a significant fraction of these are volatile organic compounds (VOCs). Factors including the life stage of the plant, temperature, environment, and stress influence the abundance and types of VOCs emitted. The analysis of VOCs released by plants during different stages or with different conditions provides insight into plant metabolism and stress responses. Collecting the VOC profiles of plants *in vivo* makes it possible to obtain a representative sample of the entire plant volatilome under controlled conditions with minimal invasiveness. In addition, *in vivo* sampling can also be used to compare the impacts of different environmental conditions or stressors on plants, i.e., the presence/absence of a pest or amount of nitrogen in soil. **Methods:** In this study, an *in vivo* plant sampling technique is introduced and validated using active sampling and thermal desorption (TD) tubes with comprehensive two-dimensional gas chromatography coupled to a time-of-flight mass spectrometer (TD-GC×GC-TOFMS). The purpose of this work is to highlight a novel technique to analyze headspace secondary plant metabolites with a minimal invasiveness. **Results:** It was concluded that *in vivo* active sampling onto TD tubes provides a wider global coverage of compounds and larger peak areas when compared to extraction by solid-phase microextraction (SPME). Additionally, the Horwitz ratio of active sampling onto TD tubes was 0.893, demonstrating this technique to be a reliable and reproducible method. Lastly, a variety of plants were sampled to assess the versatility of this technique across various plant species with different sizes and volatile profiles. Hundreds of compounds were measured with this analysis, including terpenes, aldehydes, ketones, terpenoids, and alcohols. **Conclusions:** This novel *in vivo* active sampling method provides an additional technique for extracting and analyzing volatile secondary plant metabolites.

## 1. Introduction

As plants grow, they produce many primary metabolites with a variety of functions including growth regulation, development, and reproduction [1]. Plants also produce a variety of secondary metabolites for purposes such as defense from biotic and abiotic stressors, plant communication, and the attraction of pollinators [1,2,3,4]. Volatile organic compounds (VOCs) are one of the most important groups of plant metabolites; as plants can emit them, they represent one of the few ways in which a plant can influence the behavior of other nearby organisms. For example, maize (*Zea mays*) seedlings exposed to defense VOCs from neighboring plants will produce a higher amount of volatile sesquiterpenes to protect themselves from a potential stressor, demonstrating the importance of signaling between plants [5]. In tomato (*Solanum* spp.) plants, it has been observed that some varieties release zingiberene and curcumene, which are sesquiterpenes that reduce infestation by whiteflies [6]. Sesquiterpenes are considered juvenile hormone analogs in insects that are important in insect development and reproduction [7]. There are several chemical families of plant VOCs including terpenes, ketones, aldehydes, hydrocarbons, aromatics, and thiols [8]. Understanding the VOC profile of plants provides valuable insight into plant biology, specifically in terms of plant defense to abiotic and biotic stressors, intra- and interspecies plant communication, and pollination.

There are several extraction techniques that have been used to study plant metabolites, including solvent extraction, Soxhlet extraction, and supercritical fluid extraction (SFE) [9,10,11,12,13]. Extractions can be performed on whole plants or parts of plant organs or using a sorbing matrix that has collected headspace plant VOCs [13,14,15]. Unfortunately, these methods require the use of solvents, are destructive to plants, and are time-consuming, and volatiles may be lost during the extraction process [16]. A popular and powerful approach for studying plant VOCs is using solid-phase microextraction (SPME) as the extraction mechanism [9,14,16,17,18]. SPME is a simple, solvent-free, fast, and sensitive sampling technique that can be conducted statically (no movement or flow in the headspace) or dynamically (movement/flow in the headspace), with dynamic sampling enhancing the extraction of trace compounds [19]. Although SPME has improved the analysis of plant VOCs over previous methods, this technique does have several disadvantages including complex quantification (SPME is an equilibrium method and the plant fiber partition coefficients of analytes are generally unknown), and the sorbent with its limited surface area greatly influences the quantity and type of compounds being extracted [15]. An alternative to SPME is active sampling onto a thermal desorption (TD) tube. This has been frequently used to analyze environmental, human breath, and human blood samples [20,21,22,23]. In this mode of operation, a pump pulls an air sample through a TD tube containing a bed of one or more sorbents. A larger range of chemistries, higher masses of sorbent, and a concomitant increase in surface area increase the range of analyte chemistries that can be collected and the absolute mass of analyte extracted, when compared to SPME [24,25]. Volatiles are desorbed from the TD tube by heating it while a flow of inert gas transports sorbed compounds to a smaller cold trap for focusing. Following focusing, the rapid heating of the smaller trap facilitates desorption directly into a gas chromatograph (GC) for the separation of the volatile mixture. Coupling GC with TD, as the sample introduction method, significantly improves the sensitivity, reproducibility, and recovery compared to solvent extractions and SPME [26,27]. Additionally, TD tubes can be reused approximately 100 times, samples can be stored long-term, the transportation of samples between laboratories is simplified, and the automation of TD is readily available and solvent-free [26,28,29,30].

Gas chromatography–mass spectrometry (GC-MS) is a robust and sensitive platform for plant metabolomics that can detect and identify hundreds of plant metabolites [30,31,32,33,34,35,36]. However, GC-MS has limitations such as a limited separation capacity and resolution, which results in the coelution of compounds that can lead to significant and/or less intense volatiles going unnoticed, poor quantification, and reduced mass spectral quality [37,38,39]. Comprehensive two-dimensional gas chromatography (GC×GC) drastically enhances the separation capacity and resolution compared to one-dimensional GC [37,40,41,42]. Further advancements in plant metabolomics have been made as a result of using GC×GC-MS [43,44,45]. In this field, GC×GC-MS has been used to evaluate antibacterial activities of plants, plant pathogens, and plant–pest interactions and profiling food headspace volatiles [18,46,47].

In this study, an *in vivo* active sampling method targeting plant headspace VOCs onto TD tubes was validated. As SPME is frequently used to extract plant headspace VOCs and can be implemented into the *in vivo* sampling system, some comparisons to SPME sampling were performed in order to demonstrate the different ranges and abundances of compounds collected with the two techniques. Thermal desorption with two-dimensional gas chromatography–time-of-flight mass spectrometry (TD-GC×GC-TOFMS) was used to separate and identify the VOCs emitted by various plants. Three species of plants (two varieties of tomatoes) were used in this study: *Solanum lycopersicum* (Little Napoli tomato), *Esculentum lycopersicon* (Sugar Rush tomato), *Vaccinium corymbosum* (Patriot highbush blueberry), and *Mentha villosa* (Mojito mint). These plants were chosen due to their accessibility to the research team and to highlight the versatility of the *in vivo* sampling system by collecting headspace samples from various species. Furthermore, two tomato varieties were chosen to demonstrate the robustness of the *in vivo* sampling system to differentiate volatiles from similar plants. To our knowledge, this is the first description of a reusable setup for the *in vivo* sampling of plant volatiles onto a TD tube with active sampling to collect plant VOCs using TD-GG×GC-TOFMS for the analysis. The overarching goal of this study was to provide an additional extraction technique that targets secondary plant metabolites and other headspace VOCs that allow for the analysis of a whole plant without causing damage and mimic nature as closely as possible.

## 2. Materials and Methods

### 2.1. Plants

The plants chosen for this study were Little Napoli tomato, Sugar Rush tomato, Patriot highbush blueberry, and Mojito mint. All plants were purchased from a local garden center in Edmonton, Canada. Plants were watered as needed. Mature plants were used for the collection of VOCs.

### 2.2. Thermal Desorption Tube Preparation

Standard 3″ × 0.25″ stainless steel tubes with a Tenax^®^ TA/Carbograph 1/Carbosieve sorbent bed (Markes International Ltd., Bridgend, UK) were pre-cleaned at 350 °C with a flow of 100 mL/min of ultrahigh-purity N_2_ (boil-off from liquid nitrogen Dewar) for 30 min using a TC-20 Tube Conditioning Unit (Markes International Ltd., Bridgend UK). Three-bed sorbent tubes were used for collection to maximize the chemical classes and volatile range of compounds that can be collected and analyzed. Prior to sampling, a 4 μL aliquot of internal standard solution (IS) containing 10 ng/μL of each of n-nonane-d_20_ (CDN Isotopes, Pointe-Claire, QC, Canada) and naphthalene-d_8_ (Isotec, Canton, GA, USA) in methanol (Fisher Scientific, Ottawa, ON, Canada) was spiked onto the sampling end of each TD tube using a 10 µL glass syringe (Hamilton, Reno, NV, USA). Once the internal standards were spiked, tubes were conditioned in the TC-20 at 60 °C with a flow of 50 mL/min of N_2_ for 20 min.

### 2.3. Plant Sampling System

The plant sampling system has several components, including Teflon™ tubing (McMaster-Carr, Elmhurst, IL, USA), a 64 oz. glass chamber (Richards Packaging, Winnipeg, MB, Canada), a Teflon™ plate (McMaster-Carr, Elmhurst, IL, USA), and carbon traps (Scientific Glassblowing, University of Alberta Department of Chemistry) (Figure S1) [48]. Prior to sampling the plants, the system was cleaned and dried in an oven to remove any residual impurities and to avoid carryover from previous samples. The Teflon™ tubing and plate were cleaned with 50% ethanol in water. The Teflon™ tubing and plate were dried at 150 °C for 2 h and 1 h, respectively. The 64 oz. glass chamber was washed sequentially with hexane, acetone, methanol, and water and then dried in an oven for 1 h at 150 °C. Solvents were selected due to their range in polarities to remove contaminants also with a range of polarities. All solvents were ACS-grade (Fisher Scientific, Ottawa, ON, Canada). 18.2 MΩ·cm^−1^ water from a PURELAB Dispenser (ELGA, High Wycombe, UK) was used.

The sampling system was assembled as in Figure S1. Clean air at a flow rate of 300 mL/min enters the chamber via Teflon™ tubing (1/4″ diameter) with an inline hydrocarbon trap containing activated carbon. The clean air then enters a 64 oz. glass chamber containing the plant being sampled. The glass chamber is placed inside grooves specific to the glass chamber’s dimension on top of a Teflon™ plate (Machine Fabrication, University of Alberta Department of Chemistry, Canada) that encloses the plant around its stem. Glass wool (Corning Inc., Corning, NY, USA) is used between the stem and the Teflon™ plate to close the system. Headspace air from the chamber is pulled through a second Teflon™ tube and into a TD tube using a GilAir 3 air sampling pump (Sensidyne, Clearwater, FL, USA) calibrated to 50 mL/min, as recommended by the Environmental Protection Agency (EPA) [23]. The selection of materials for the sampling system was based on minimal sorption and leaching capabilities, ease of cleaning, cost, and availability. Prior to the thermal desorption of the samples, collected plant samples underwent an initial dry purge step to remove water from the tube without removing trapped VOCs. The dry purge was conducted using the TC-20 unit at a temperature of 50 °C for 10 min with a nitrogen flow of 50 mL/min. Tubes were then capped immediately and kept at 4 °C until analysis. In this study, the whole plant, excluding the soil, was sampled.

### 2.4. Thermal Desorption Method

Samples were desorbed using a TD100-xr thermal desorption unit (Markes International Ltd., Bridgend, UK). The following conditions were used: desorption flow, 50 mL/min, splitless flow; desorption temperature, 250 °C; desorption time, 25 min; cold trap trapping temperature, −25 °C; cold trap desorption temperature, 320 °C; and a cold trap desorption split ratio of 1:200.

### 2.5. SPME Method

A three-phase fiber consisting of divinylbenzene/carbon wide-range/polydimethylsiloxane (DVB/CAR/PDMS) (Supelco, Bellefonte, PA, USA) was conditioned for 10 min at 270 °C prior to sampling each plant. For sample collection, the fiber was exposed to the plant system through the base. The extraction was for 1 h at room temperature followed by immediate manual injection in the GC inlet at 250 °C for 5 min with a split ratio of 1:200.

### 2.6. GC×GC-FID/TOFMS Method

An Agilent 7890A GC with an FID (Agilent Technologies, Santa Clara, CA, USA) equipped with an INSIGHT flow modulator (SepSolve Analytical, Waterloo, ON, Canada) and Markes BenchTOF Select (Markes International Ltd., Bridgend, UK) mass spectrometer was used for the analysis. The first-dimension column used was a 30.0 m × 0.25 mm, 0.25 µm film thickness Rtx-5 column (Chromatographic Specialties, Brockville, ON, Canada). The second-dimension column was a 5.0 m × 0.25 mm, 0.25 μm film thickness Rtx-17 (Chromatographic Specialties, Brockville, ON, Canada). Helium (5.0 grade; Linde, Burr Ridge, IL, USA) was used as the carrier gas. The GC oven program was 50 °C (3 min), followed by an 8 °C/min ramp to 280 °C (4 min). Carrier gas flows were set to 0.73 mL/min in ^1^D and 15.0 mL/min in ^2^D with a modulation period of 2.2 s with a flush time of 100 ms, and the modulator operated in reverse fill-flush mode. After the ^2^D separation, a purged microfluidic splitter (Markes International Ltd., Bridgend, UK) was used to split the flow between the FID and the MS. The MS and FID transfer lines were the deactivated fused silica of dimensions 2.5 m × 0.18 mm and 5 m × 0.32 mm, respectively. The hydrogen flow for the FID was 30 mL/min, the air flow was 400 mL/min, and the helium makeup flow was 20 mL/min. The MS used electron ionization at 16 eV and 70 eV in tandem with a *m*/*z* range of 40–600. The data acquisition rates of the Bench-TOF and the FID were set to 100 Hz.

### 2.7. Data Analysis

GC×GC-TOFMS data were processed using GC Image GC×GC Software, version 2022r1 (GC Image LLC, Lincoln, NB, USA). For qualitative analysis, the following parameters were used: smoothing: 0.4 and 0.1 for ^1^D and ^2^D, respectively; sensitivity and minimum differentiation interval: 5; minimum area: 75; minimum volume: 65; and minimum peak: 10. The NIST Mass Spectral Library (NIST 17, version 2.4) was used to match mass spectral data obtained from individual peaks. A standard mix containing 19 terpenoid compounds was used for positive identification. The mass spectra and retention indices were compared to the 19 terpenoid standard compounds. The standard was prepared by spiking 4 μL of a 5 ng/μL solution of cannabis terpenes standard #1 (Restek, Corp., Bellefonte, PA, USA) diluted in methanol (Fisher Scientific, Ottawa, ON, Canada) onto a thermal desorption tube and analyzed as described in Section 2.2.

## 3. Results

### 3.1. Active Sampling onto a TD Tube vs. SPME

**Figure 1:**
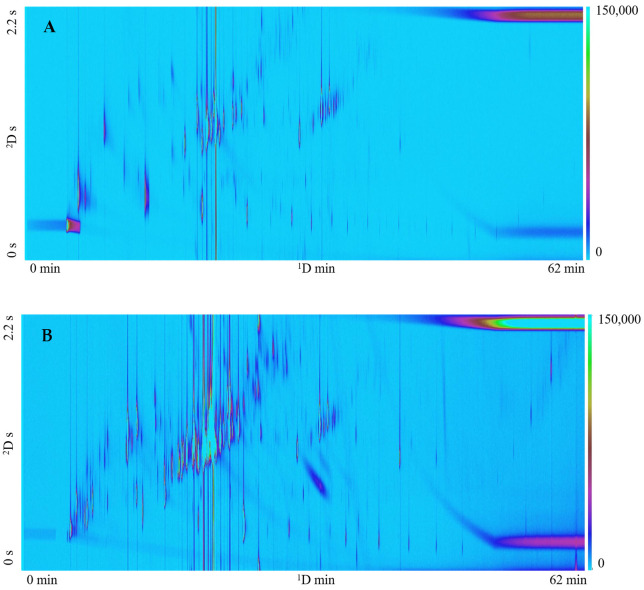
GC×GC-TOFMS TIC contour plots obtained from Little Napoli tomato plant. (**A**) SPME extraction. (**B**) Active sampling onto a TD tube extraction.

A comparison between SPME and active sampling onto a TD tube was conducted using Little Napoli tomato, Patriot blueberry, and Mojito mint. Representative total ion chromatograms (TICs) obtained from Little Napoli tomato with each sampling method are provided in Figure 1. Comparisons between extraction techniques were made based on the number of tentatively identified compounds and their relative peak areas (Table 1). Comparisons for Mojito mint and Patriot blueberry bush can be found in Figure S2. An individual peak comparison between the two sampling methods for the three plants is presented in Table 2. 

### 3.2. Reproducibility of the In Vivo Plant Sampling System Using Active Sampling and TD-GC×GC-TOFMS

The reproducibility of the plant sampling system was assessed using the Horwitz ratio. In order to perform the calculations, the intensity of the manually spiked IS from three replicate injections was used.

### 3.3. In Vivo Active Sampling Using Multiple Plant Species

**Figure 2:**
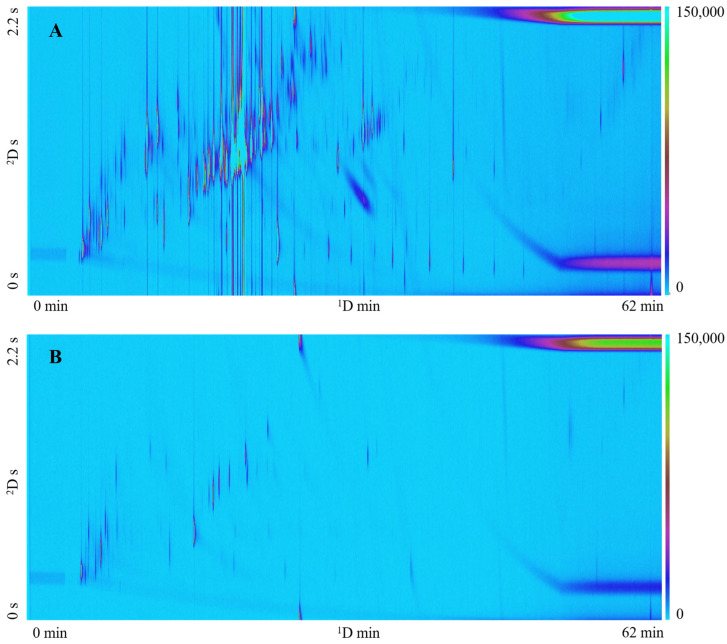

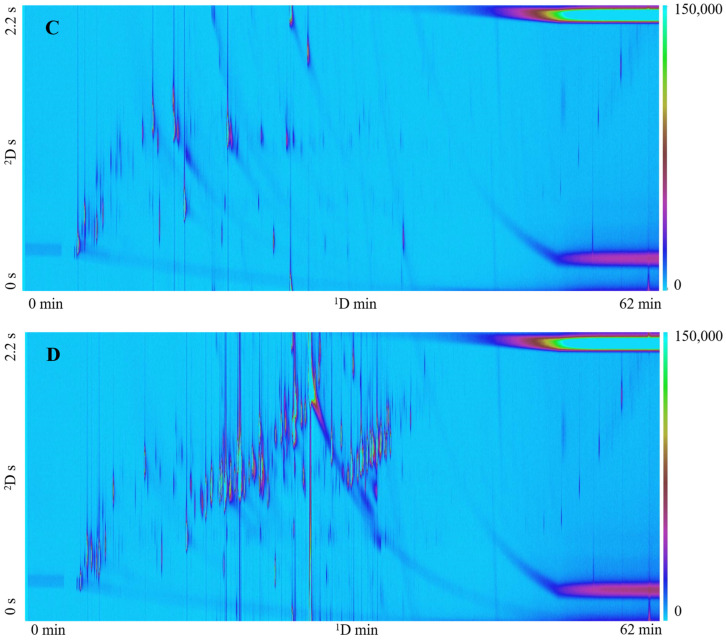
GC×GC-TOFMS TIC contour plots of four plant species using active sampling onto a TD tube. (**A**) = Little Napoli tomato, (**B**) = Sugar Rush tomato, (**C**) = Patriot blueberry bush, (**D**) = Mojito mint.

To demonstrate the applicability of this technique across a variety of plants, four plant species were sampled using active sampling onto a TD tube: Little Napoli tomato, Sugar Rush tomato, Patriot blueberry bush, and Mojito mint. Figure 2 presents typical chromatograms for each plant sampled using this approach. The chromatograms exhibit a wide range of compounds including monoterpenes, sesquiterpenes, benzenoids, alcohols, and aldehydes; some of the compounds tentatively identified in the samples are listed in Table 2.

**Table 2:** Selected VOCs captured by the *in vivo* sampling system and their corresponding retention times, retention indices, and peak areas.

Plant	Compound	^1^t_R_ (min)	^2^t_R_ (s)	Exp RI	Library RI	MS Match Factor	SPME Peak Area	TD Tube Peak Area
Mojito Mint	D-Carvone	27.83	1.82	1273	1246	916	1.06 × 10^9^	1.00 × 10^9^
	Eucalyptol	21.05	1.2	1048	1032	899	1.45 × 10^7^	1.40 × 10^8^
	Limonene *	20.9	1.07	1044	1030	917	7.47 × 10^7^	2.49 × 10^8^
	cis-Dihydrocarvone	26.33	1.66	1219	1195	878	6.89 × 10^7^	6.80 × 10^7^
	(-)-Dihydrocarvyl acetate	29.92	1.31	1349	1330	856	1.08 × 10^7^	2.06 × 10^7^
Little Napoli	γ-Terpinene *	21.12	1.11	1051	1060	800	5.28 × 10^8^	1.64 × 10^9^
	3-Carene *	19.98	1.02	1016	1011	N/A	1.06 × 10^8^	6.49 × 10^8^
	Camphene *	17.64	0.94	947	952	N/A	N.D.	5.30 × 10^7^
	Hexanal	12.72	1.25	807	801	709	1.32 × 10^6^	2.06 × 10^7^
	Humulene *	33.70	1.34	1494	1454	N/A	1.83 × 10^7^	1.87 × 10^7^
Patriot Blueberry	Cyclohexanol	14.74	1.26	865	880	672	1.24 × 10^7^	2.20 × 10^7^
	2-Ethyl-2-butenal	12.65	1.33	805	801	670	7.16 × 10^6^	1.83 × 10^7^
	2-Hexen-1-ol (E)	15.11	1.23	874	868	705	3.20 × 10^6^	7.96 × 10^6^
	4-Hexenyl acetate	19.95	1.22	1015	1020	895	2.87 × 10^7^	3.31 × 10^7^
	Benzaldehyde	18.63	2.13	976	962	607	6.11 × 10^5^	1.63 × 10^6^
Sugar Rush	α-Pinene *	18.48	0.96	972	978	N/A	N/A	4.43 × 10^6^
	2-Carene	19.07	1.06	989	1001	730	N/A	2.72 × 10^6^
	α-Terpinene *	20.09	1.12	1020	1017	N/A	N/A	1.49 × 10^6^

^1^t_R_: first-dimension retention time measured in minutes. ^2^t_R_: second-dimension retention time measured in seconds. RI: retention index. Compounds selected based on uniqueness to the respective plant and/or being an abundant compound in the chromatogram. * = positively identified with a terpene standard mix.

## 4. Discussion

In this work, a reusable *in vivo* plant sampling system based on active sampling onto a TD tube is described. This system permits the analysis of whole, living plants. SPME has frequently been used to sample the headspace of plants [9,14,16,17,18]. However, SPME has limitations, such as a limited surface area and a bias simultaneously against both highly volatile and heavier semi-volatile peaks [15]. It must be noted that plant identifications were based upon the identification tags provided by the garden center from which the plants were purchased. Consequently, we do not know the exact variety of tomato, mint, and blueberry that were used. However, from a point of view of demonstrating the methodology, the exact varieties of the plants are not important.

This approach can be adapted to plants of varying sizes by altering the sizes of the glass chamber and Teflon™ plate and potentially adjusting the flow of air supplied to the plant. For these experiments, it was observed that plants became visibly distressed (drooping leaves) when lower supply air flows were used. Reasons for the plant stress could include the accumulation of oxygen in the chamber or a decreased supply of CO_2_. A further stressor could also be the accumulation of plant VOCs in the atmosphere of the chamber. Increasing the air flow to 300 mL/min appeared sufficient to keep the plants content within the sampling chamber. Users must be aware of this parameter to avoid stressing plants and biasing results. The system can also potentially be adapted for other plant sampling scenarios to target specific plant organs by adjusting which parts of the plant are isolated from the surrounding environment (e.g. flowers, leaves, fruits, soil, etc.). For targeting specific classes of compounds of plant volatiles, users can select sorbent beds for the TD tubes that will best trap the compounds of interest. 

### 4.1. TD Tube vs. SPME

The total number of peaks observed using both techniques are similar for Mojito mint, whereas for Patriot blueberry, SPME detected more compounds, and active sampling captured more compounds than SPME for Little Napoli tomato. Across all three plants, there was an increase in TPA when using active sampling onto a TD tube. The increase in peak area with TD sampling is evident. This is due to the more exhaustive extraction with a higher absolute recovery of volatiles (inherent to active sampling) for the TD method than for SPME [19,24]. Additionally, SPME generally works well for moderately volatile compounds—those with sufficient volatility to end up in the gas phase, while not being so volatile that they sorb very weakly to the fiber. Figure 1 shows how the TD approach provides better chromatography and analyte recovery in the early portion of the chromatogram. This is attributed to the narrower injection band from the TD instrument, which provides focused injection of all analytes including early-eluting ones. With SPME, early-eluting peaks are not effectively focused at the head of the GC column due to their relatively high volatility and low retention factor on the column, an issue not easily remedied with a typical GC-MS system. Generally, active sampling onto TD tubes was able to capture the highly volatile compounds with a low molecular mass and the low volatile compounds with a larger molecular mass compared to SPME. Compounds that overlapped between SPME and active sampling were the semi-volatile compounds. However, as mentioned previously, the compounds that were present in both techniques had a larger peak area when collected with active sampling onto TD tubes. Although the overloading of the chromatographic column can occur for very abundant compounds when using active sampling onto TD tubes (as observed in the chromatograms for Little Napoli tomato and Mojito mint), this can be corrected by optimizing the column loading (by means of the split ratio and/or sampling flows and sampling times). Optimizing these parameters would be sample and instrument-specific. When working with natural products, it is not uncommon for multiple injections to be performed for each sample: one low-split injection to characterize minor constituents and one high-split injection for the major constituents. With TD, this can be easily achieved by either collecting multiple samples in parallel from each plant or, if instrumentation permits, splitting injections from a single tube and recollecting the fraction of the sample that is not directed to the GC-MS system. Finally, it must also be noted that transporting multiple samples long distances from a sampling location (e.g., field site or greenhouse) to the laboratory is relatively trivial with TD tubes. This is much more difficult with analytes on SPME fibers, highlighting one final advantage of active sampling onto sorbent tubes over SPME when it comes to *in vivo* plant metabolomics studies, as has been noted previously [24].

### 4.2. Reproducibility of the In Vivo Plant Sampling System

To be useful, a sampling method must be reproducible across multiple analyses of the same sample. Validating the extraction reproducibility of the *in vivo* plant sampling system using active sampling onto TD tubes, even with the same plant and sequential collections, is difficult, as the plant VOCs are released dynamically and concentrations may change over the period of time while the samplings are conducted. A criterion accepted by AOAC International for measuring reproducibility is to evaluate the Horwitz ratio of the analytical method [49]. For this work, we elected to use the manually spiked IS with a known concentration (C) and measured the observed relative standard deviation (RSD_R_) based on the average peak area from three measurements because, as previously mentioned, plant VOC concentrations can change rapidly and a known concentration is required for the Horwitz calculations. The predicted relative standard deviation (PRSD_R_) was calculated using the Horwitz equation (Equation (1)) [49]. The Horwitz ratio can then be calculated following Equation (2) [49]. In this experiment, the RSD_R_ and PRSD_R_ of the internal standard were 9.70% and 10.9%, respectively. The methods obtaining a Horwitz ratio value between 0.5 and 2 are considered precise analytical methods [49]. For the *in vivo* active sampling method, a Horwitz ratio of 0.893 was obtained (see detailed calculations in the Supplemental Information), deeming this extraction technique a precise analytical method.

PRSD_R_(%) = 2C^−0.15^ (1)

(2) $$Horwitz\;Ratio=\frac{{RSD}_{R}}{{PRSD}_{R}}$$

### 4.3. Validation of In Vivo Active Sampling Using Multiple Plant Species

As displayed in Figure 2, the *in vivo* active sampling methodology followed by TD is applicable to a wide range of plant species and can likely be applied to most plant species, demonstrating its versatility. Monoterpenes, sesquiterpenes, benzenoids, alcohols, and aldehydes were some of the many classes detected using the *in vivo* plant sampling system (Table 2). The compounds highlighted in Table 2 were selected as they are known to play significant roles in plant defense and communication. For example, terpenes such as limonene attract pollinators, and α-pinene can repel pests [50]. Benzaldehyde is known to be involved in various plant functions performing as a pollinator attractant and an antifungal compound [51]. Furthermore, C_6_-aldehydes and hexenyl acetate greatly influence insect–plant interactions and play valuable roles in plant defense [52]. 

The compounds collected by the *in vivo* active sampling system are comparable to other currently used techniques. For example, the essential oil extraction of Mojito mint leaves followed by liquid injection yields limonene and eucalyptol, which are both present in high abundances using the *in vivo* active sampling system [53,54]. SPME-GC-MS analysis of homogenized blueberry samples detected several volatile compounds, including benzaldyde and 2-hexen-1-ol (E), which are both detected when sampling *in vivo* [55]. In another study conducted by Namrocka et al. using SPME-GC×GC-TOFMS, they detected several of the same compounds from Little Napoli tomato plant leaves as the technique described in this paper, including hexanal and γ-terpinene [56]. Additionally, the results observed from collecting headspace plant volatiles using the described *in vivo* plant sampling system for Sugar Rush tomato are comparable to those found by Zhang et al. by purging and trapping on Tenax TA resin followed by solvent elution [57].

### 4.4. General Discussion

Ultimately, it was deemed that the use of active sampling with TD tubes outperforms SPME in terms of the overall VOC coverage and recovered amounts of plant volatiles. The use of active sampling with TD tubes presents other advantages as well, including an easier quantification of target analytes due to the removal of the differential and unknown uptake rates of compounds by SPME, and the ease with which TD tubes can be transported and stored for the long term [28,29].

One of the largest benefits of the *in vivo* active sampling technique is the ability to sample whole plants without destruction. Through keeping the plant alive, it becomes an enhanced model for how the plant naturally exists and can therefore provide a more accurate profile of plant VOC production with minimal stress (or when specific experimental stressors are applied to the plant, e.g., changes in nutrients, the presence of a pest, etc.). This system can be used for several applications to analyze plant volatiles. With the human population growing rapidly, there is concern for food shortages, especially those caused by insect pests [58]. Thankfully, there are plants that can naturally deter insect herbivores by releasing VOCs, including wheat, maize, and tomato species [5,6,48,59,60]. To further understand the relationship between plant volatiles and pests, this *in vivo* sampling system can be utilized to characterize plant headspace volatiles that may be responsible for pest deterrence by collecting samples with and without exposures to pests or by characterizing different varieties of the same plant species with known deterrent properties. Another application of the *in vivo* sampling system is in identifying compounds responsible for pollinator attraction. Understanding how plants are being pollinated and by what species could enhance current agricultural practices, as certain volatiles attract specific classes of pollinators, which can drastically influence the reproductive success of plants. Other areas of research that this system can be applied to is studying the interactions between intra- and interplant species, monitoring the production of VOCs throughout the life cycle of a plant, assessing the effects of environmental or physical stressors on plants, analyzing genetic modifications, adapting the system to collect plant VOCs at different locations, and several other fields. With the use of the *in vivo* plant sampling system, plant biology and chemistry can be further understood across several plant species.

## 5. Conclusions

The technique of using *in vivo* active sampling for collecting plant VOCs is a reliable and reproducible method that can be used for many plant species. In addition, using active sampling with TD rather than SPME provides a wider global coverage of VOCs with higher intensities, aiding in compound identification and quantification, making it an effective tool for plant analysis. Additionally, by relying on TD tubes, it is much easier to transport samples between sampling locations and the laboratory compared to using SPME, and samples can be stored long term until analysis. Coupling *in vivo* active sampling to TD-GC×GC-TOFMS further enhances the ability to separate, detect, and identify several hundreds of VOCs simultaneously. There are several fields of research that can utilize this *in vivo* active sampling method to further understand plant biology and chemistry, including plant–pest and plant–pollinator interactions, exemplifying the importance of this technique. 

## Figures and Tables

**Table 1:** Comparison of SPME and active sampling onto TD tubes.

Plant	TPA SPME	TPA TD Tube	Number of Peaks SPME	Number of Peaks TD Tube
Little Napoli	1.01 × 10^9^ *	5.13 × 10^9^ *	138 *	283 *
Patriot Blueberry	1.45 × 10^8^	1.66 × 10^8^	145	92
Mojito Mint	1.87 × 10^9^	2.74 × 10^9^	266	252

* A tomato fruit was present during sampling time. TPA = total peak area.

## Data Availability

Data can be found in the Supporting Information documents.

## Supplementary Materials

The following supporting information can be downloaded at: https://www.mdpi.com/article/10.3390/metabo14110623/s1, Figure S1: *in vivo* active plant sampling system; Figure S2: GC×GC-TOFMS TIC contour plot of clean *in vivo* plant sampling system. Page 3: Horowitz Calculation.
