# Supplementary material for: Analysis of In Vivo Plant Volatiles Using Active Sampling and TD-GC×GC-TOFMS
Source: Metabolites. 2024 Nov 14;14(11):623. doi: 10.3390/metabo14110623 (PMC11596084; doi:10.3390/metabo14110623)
Supplement: Supplementary file 1 [file metabolites-14-00623-s001.zip › SupplementaryInformation.pdf]

Electronic Supplementary Information

Analysis of *in vivo* Plant Volatiles Using Active Sampling and TD-GC×GC-TOFMS

Sheri A. Schmidt[1], *Ewenet Y. Mesfin*[1], *Chaminda De Silva Weeraddana*[2], *A. Paulina de la Mata*[1], *Alejandro C. Costamagna*[2], *James J. Harynuk*[1]

[1]Department of Chemistry, University of Alberta; [2]Department of Entomology, University of Manitoba

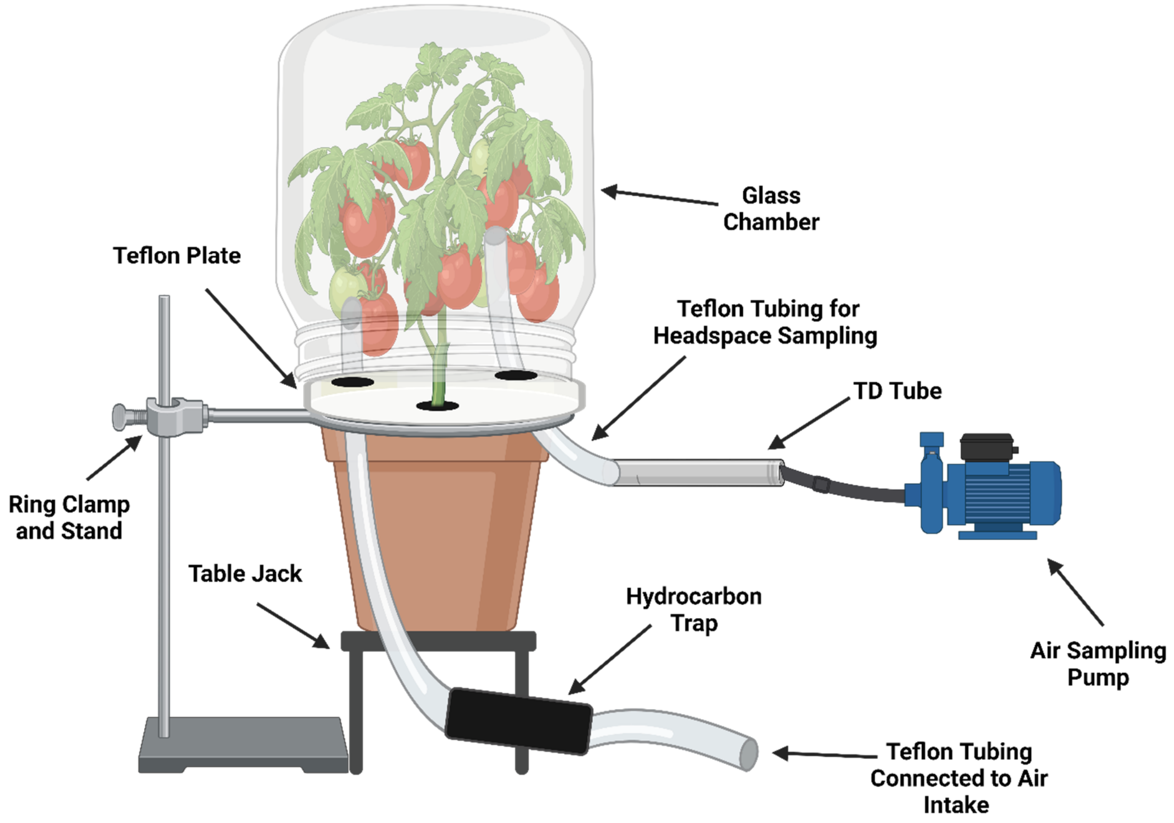

Figure S1. *in vivo* active plant sampling system with a thermal desorption tube.

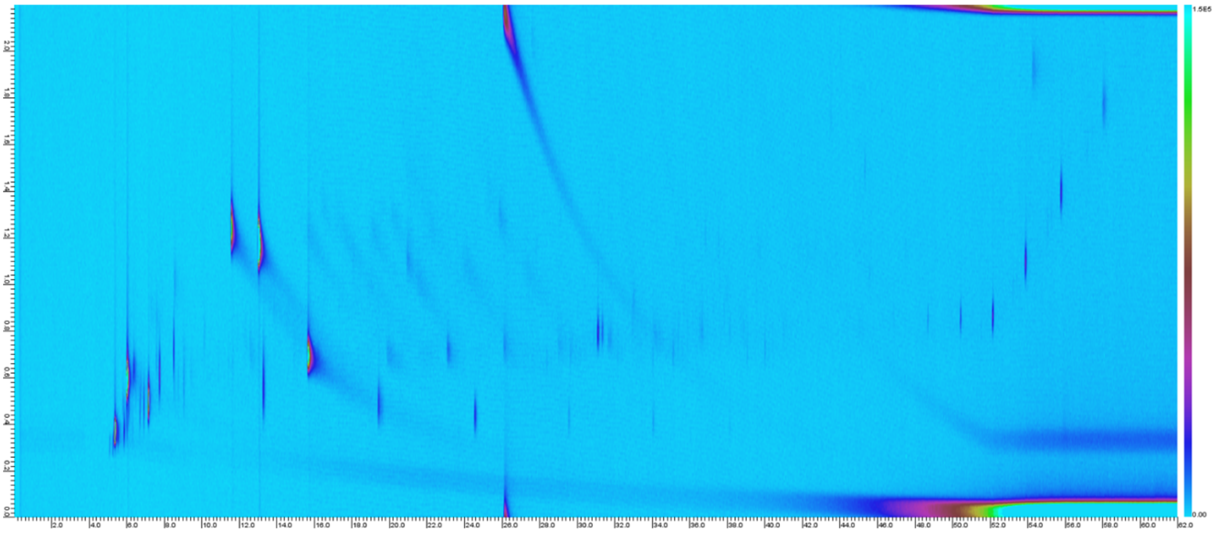

Figure S2. GC×GC-TOFMS TIC contour plot of the clean *in vivo* plant sampling system.

Horwitz Calculation

A solution containing n-nonane-d_{20} was used to estimate the precision of the method based on the Horwitz Ratio. A solution of n-nonane-d_{20} diluted in methanol was prepared at a concentration of 10 ng/µL. 4µL of this solution was manually spiked onto a thermal desorption tube using a 10 µL syringe. The Horwitz Ratio calculation requires the predicted relative standard deviation ($PRSD_R$), which is calculated based on analyte concentration (*C*), expressed as a dimensionless w/w ratio.

$$PRSD_R(\%) = 2C^{-0.15}$$

Calculation of *C*, based on the concentration of n-nonane-d_{20} and the density of methanol at the laboratory temperature proceeds as follows:

for n-nonane-d_{20}:

$$\left(\frac{10\ ng}{1.0\ \mu L}\right) \times (4.0\ \mu L) \times \left(\frac{1\ g}{10^{9}\ ng}\right) = 4.0 \times 10^{-8} g$$

for methanol:

$$\left(0.7913 \frac{g}{mL}\right) \times (4.0\ \mu L) \times \left(\frac{1\ mL}{10^{3}\ \mu L}\right) = 3.1652 \times 10^{-3} g$$

thus,

$$C = \frac{4.0 \times 10^{-8}\ g}{3.1652\ \times 10^{-3}\ g} = 1.2637 \times 10^{-5}$$

and,

$$PRSD_R(\%) = 2(1.2637 \times 10^{-5})^{-0.15}\ = 10._{9}\,\%$$
